# Application of a spatially-weighted Relief algorithm for ranking genetic predictors of disease

**DOI:** 10.1186/1756-0381-5-20

**Published:** 2012-12-03

**Authors:** Matthew E Stokes, Shyam Visweswaran

**Affiliations:** 1 Department of Biomedical Informatics and the Intelligent Systems Program, University of Pittsburgh, 5607 Baum Boulevard, Pittsburgh, PA, 15206, USA

**Keywords:** Feature selection, Relief, Single nucleotide polymorphisms, Genetic interactions, Epistasis

## Abstract

**Background:**

Identification of genetic variants that are associated with disease is an important goal in elucidating the genetic causes of diseases. The genetic patterns that are associated with common diseases are complex and may involve multiple interacting genetic variants. The Relief family of algorithms is a powerful tool for efficiently identifying genetic variants that are associated with disease, even if the variants have nonlinear interactions without significant main effects. Many variations of Relief have been developed over the past two decades and several of them have been applied to single nucleotide polymorphism (SNP) data.

**Results:**

We developed a new spatially weighted variation of Relief called Sigmoid Weighted ReliefF Star (SWRF*), and applied it to synthetic SNP data. When compared to ReliefF and SURF*, which are two algorithms that have been applied to SNP data for identifying interactions, SWRF* had significantly greater power. Furthermore, we developed a framework called the Modular Relief Framework (MoRF) that can be used to develop novel variations of the Relief algorithm, and we used MoRF to develop the SWRF* algorithm.

**Conclusions:**

MoRF allows easy development of new Relief algorithms by specifying different interchangeable functions for the component terms. Using MORF, we developed a new Relief algorithm called SWRF* that had greater ability to identify interacting genetic variants in synthetic data compared to existing Relief algorithms.

## Background

Correlating genetic variants with disease is the primary method used for elucidating the genetic causes of common diseases like Alzheimer’s disease and type 2 diabetes mellitus. Compared to common diseases, the genetic underpinnings of Mendelian diseases such as cystic fibrosis and thalassemia are generally simpler, as they are caused by variation at a single genetic locus. In contrast, common diseases are caused by genetic variants at multiple loci, with each locus conferring modest risk of developing disease. The most common genetic variation is the single nucleotide polymorphism (SNP), which is a DNA sequence variation occurring when a single base pair at a specific location in the genome differs among individuals in a population. Due to the rapidly falling cost of genotyping, the volume of genomic data available to researchers is growing very quickly. A key challenge in the analyses of such data is to develop computational methods that efficiently identify complex associations between genetic variants (such as SNPs) and disease [1].

One example of a complex interaction among genetic variants is *epistasis*, wherein the genetic variants interact in a nonlinear fashion in their association with disease. Epistatic effects are particularly difficult for many algorithms to detect, because they may require examination of interacting genetic variants taken together, rather than one at a time. Because the number of potential interactions grows exponentially with the number of variants being examined, it is not computationally feasible to evaluate every potential higher order interaction in high-dimensional data. Many current methods address this problem by using a two stage procedure where they identify a subset of variants associated with disease in a univariate fashion in the first stage, and then evaluate interactions only within this smaller set of variants in the second stage [2]. However, if epistatic variants have small or no main effects, the univariate selection in the first stage will exclude the epistatic variants so that they are not even considered in the second stage. One approach to overcoming this weakness is to use methods in the first stage that will identify variants with main effects, interaction effects, and both main and interaction effects. The Relief algorithms are an excellent candidate for application in the first stage since it is capable of identifying variants with both main and interaction effects.

The Relief algorithms are a family of attribute weighting algorithms that can efficiently identify associations between attributes (e.g., SNPs) and the class (e.g., disease status) even if the attributes have nonlinear interactions (e.g., epistatic) without significant main effects [3,4]. These algorithms run in time *O*(*n*^*2*^*a*), where *n* is the number of samples and *a* is the number of attributes. For SNP data, typically *a* >>*n*, making Relief algorithms particularly attractive for this domain. In the next section, we briefly describe the original Relief algorithm, and in the section after that we describe extensions of Relief that have been applied to SNP data.

### Relief

The Relief algorithm was first described by Kira and Rendell [3] as a simple, fast, and effective approach to attribute weighting. The output of the Relief algorithm is a weight between −1 and 1 for each attribute, with more positive weights indicating more predictive attributes. The pseudocode for Relief is shown below. The weight of an attribute is updated iteratively as follows. A sample is selected from the data, and the nearest neighboring sample that belongs to the same class (*nearest hit*) and the nearest neighboring sample that belongs to the opposite class (*nearest miss*) are identified. A change in attribute value accompanied by a change in class leads to upweighting of the attribute based on the intuition that the attribute change could be responsible for the class change. On the other hand, a change in attribute value accompanied by no change in class leads to downweighting of the attribute based on the observation that the attribute change had no effect on the class. This procedure of updating the weight of the attribute is performed for a random set of samples in the data or for every sample in the data. The weight updates are then averaged so that the final weight is in the range [−1, 1]. The attribute weight estimated by Relief has a probabilistic interpretation. It is proportional to the difference between two conditional probabilities, namely, the probability of the attribute’s value being different conditioned on the given nearest miss and nearest hit respectively [5].

Relief Algorithm

set *W*[*a*] = 0 for each attribute *a*

for *i* = 1 to *n* do

select sample *s*_*i*_ from data at random

find nearest hit *s*_*h*_ and nearest miss *s*_*m*_

for each attribute *a* do

Δ*W*_*i*_[*a*] = *diff*(*a*, *s*_*i*_, *s*_*m*_) - *diff*(*a*, *s*_*i*_, *s*_*h*_)

*W*[*a*] = *W*[*a*] + Δ*W*_*i*_[*a*]

end for

end for

for each attribute *a* do

*W*[*a*] = *W*[*a*] / *n*

end for

where *diff*(*a*, *s*_*i*_, *s*_*j*_) = 0, if *s*_*i*_[*a*] = *s*_*j*_[*a*]

= 1, if *s*_*i*_[*a*] ≠ *s*_*j*_[*a*]

### ReliefF, SURF, and SURF*

The ReliefF algorithm extends Relief to deal with data that is noisy, incomplete, and has more than two classes. The original Relief algorithm uses 2 neighboring samples (1 *nearest hit* and 1 *nearest miss*) during each iteration of the outer *for* loop in the pseudocode above. ReliefF uses *2k* neighboring samples (*k* nearest hits and *k* nearest misses), and averages their contributions to update the attribute weights [6]. In contrast to most other feature ranking or feature selection methods that consider attributes univariately, Relief algorithms are able to capture attribute interactions because the global distance measure which defines sample proximity is a multivariate function. However, because the nearest neighbors are identified by a distance measure that incorporates all attributes, the presence of many irrelevant or noisy attributes (as in SNP data) can lead to suboptimal identification of nearest neighbors.

In ReliefF, as the number of nearest neighbors of each class (parameter *k*) is varied, a trade-off occurs. The addition of more neighbors decreases the algorithm’s susceptibility to noise. However, the inclusion of more neighbors leads to decreased ability in identifying locally interacting attributes. In ReliefF, the number of nearest neighbors used is a user-specified parameter that is constant from iteration to iteration. Spatially sensitive variations of ReliefF, on the other hand, use a neighborhood of constant spatial dimensions, rather than a constant number of neighbors. In densely populated regions of the sample space, more nearby neighbors are used, improving the discriminative power of the algorithm. Conversely, in sparsely populated regions of the sample space, fewer neighbors are used to update the attribute weights. This improves performance because distant uninformative neighbors are not used in order to meet a *k* neighbor quota, as in ReliefF [7].

The most recently described spatially sensitive methods are Spatially Uniform ReliefF (SURF) and SURF* (called “SURF star”). SURF uses all neighbors found within a certain distance threshold to update the attribute weights. SURF* uses both nearby and distant neighbors to update the attribute weights. Nearby neighbors are used to update the attributes weights in the same way as SURF. In addition, SURF* uses the distant neighbors to update the attribute weights in a manner opposite to the weight update rule used for the nearby neighbors. SURF* has been applied to synthetic genomic data and has been shown to be more accurate than ReliefF and SURF in identifying interacting genetic variants [8].

Iterative variations of ReliefF exist, and some have greater accuracy than the corresponding non-iterative variations [9]. Tuned ReliefF (TuRF), for example, reduces the effect of noisy attributes by dropping the least predictive attributes from the dataset and running ReliefF iteratively [10]. Because TuRF essentially runs ReliefF multiple times, improvement of the underlying algorithm will improve the iterative results. As such, we focus our attention on non-iterative variations of Relief.

In this paper, we formulate a new variation of Relief called Sigmoid Weighted ReliefF Star (SWRF*). We evaluate SWRF* on synthetic data and compare its performance to that of ReliefF and SURF*. We also present a framework called the Modular Relief Framework (MoRF) that can be used to develop novel variations of Relief. MoRF facilitates the formulation of new variations of Relief by allowing the specification of new functions for component terms. The SWRF* algorithm was developed using the MoRF framework. In contrast to previous variations of Relief, SWRF* utilizes a soft neighbor weighting kernel.

## Results and discussion

Our work generated two main results. First, we present the MoRF framework, which can specify different variations of Relief by using component functions. Next, we use MoRF to formulate SWRF* and we evaluate SWRF* on synthetic genomic datasets.

### Modular Relief Framework (MoRF)

We now describe a framework that abstracts the common features of the Relief algorithms. This framework has several component terms; a distinct specification of each of these terms leads to the formulation of a distinct variation of Relief. The output is a weight for each attribute, which is computed iteratively over samples. The change in the weight for attribute *a* based on sample *s*_*i*_is computed as:

(1) $$\Delta W_{i}\left[a\right]=\frac{\sum_{s_{j}\in S}c\left(s_{i},s_{j}\right)\text{diff}\left(a,s_{i},s_{j}\right)f\left({\text{dist}}_{\mathrm{ij}}\right)}{\sum_{s_{j}\in S}\left|f\left({\text{dist}}_{\mathrm{ij}}\right)\right|}$$

where ∆*W*_*i*_[*a*] is the change in the weight for attribute *a* based on sample *s*_*i*_. The final weight *W*[*a*] is simply the average of the individual updates, taken over a set of samples *S*. That is, $W\left[a\right]=\frac{1}{n}\sum_{i=1}^{n}\Delta W_{i}\left[a\right]$ where *n* is the cardinality of *S*. The set *S* may constitute the entire dataset or a randomly selected subset of the dataset. We also have:

(2) $$c\left(s_{i},s_{j}\right)=\{\begin{array}{c} -1\;\text{if}\;\text{class}\left(s_{i}\right)=\text{class}\left(s_{j}\right)\\ 1\;\text{if}\;\text{class}\left(s_{i}\right)\neq \text{class}\left(s_{j}\right) \end{array}$$

(3) $$\text{diff}\left(a,s_{i},s_{j}\right)=\{\begin{array}{c} 0\;\text{if}\;s_{i}\left[a\right]=s_{j}\left[a\right]\\ 1\;\text{if}\;s_{i}\left[a\right]\neq s_{j}\left[a\right] \end{array}$$

(4) $${\text{dist}}_{\mathrm{ij}}=\sum_{a}\text{diff}\left(a,s_{i},s_{j}\right)$$

Where *class*(*s*_*i*_) is the class of sample *s*_*i*_ and *s*_*i*_[*a*] is the value of attribute *a* in sample *s*_*i*_. In Equation 1, *f*(*dist*_*ij*_) is a neighbor weighting function that assigns a weight to a sample *s*_*j*_ based on its distance from the sample *s*_*i*_. The role of the sum in the denominator in is to normalize the weight update so that *W*[*a*] is in the range [−1, 1]. In the following sections, we describe each term in Equation 1 in greater detail.

#### Class comparator

The class comparator, denoted by *c*(*s*_*i*_,*s*_*j*_) and defined in Equation 2 is used to compare the class values of samples *s*_*i*_ and *s*_*j*_. It expresses the fact that Relief uses attribute mismatches between a sample and a neighbor in arithmetically opposite ways depending on if the neighbor is of the same class (a *hit*) or of a different class (a *miss*).

#### Local difference function

The difference function, denoted by *diff* (*a, s*_*i*_*, s*_*j*_), is used to determine the effect of a change along a single dimension (attribute). For nominal attributes such as SNPs, a simple match-mismatch *diff* function is used as defined in Equation 3. The difference between samples *s*_*i*_ and *s*_*j*_ along dimension *a* is 0 if *s*_*i*_[*a*] and *s*_*j*_[*a*] have the same values, and 1 if the values are different. However, other possibilities for the *diff* function can be considered. For example, samples *s*_*i*_and *s*_*j*_ are defined to be equal if both samples have one or more copies of the minor allele for attribute *a*, which corresponds to the dominant genetic model at locus *a*. The *diff* function can be defined in a similar fashion to represent the recessive or the heterozygote genetic model.

#### Global distance function

In order to locate sample neighbors, Relief uses a distance measure, denoted by *dist*_*ij*_. The commonest distance measure for samples with nominal attributes such as SNP data is the Hamming distance, which is defined as the number of attributes at which two samples differ in value, and is shown in Equation 4. Equation 4 also shows that the Hamming distance is simply a sum of the *diff* function over all attributes. In general, *dist*_*ij*_ need not be defined in terms of *diff*, and other possibilities for the global distance function can be considered for SNP data. For example, if SNP values are coded as 0, 1 or 2 (for 0, 1 or 2 copies of the minor allele), the taxicab distance is defined as follows:

$${\text{dist}}_{\mathrm{ij}}=\sum_{a}\left|s_{i}\left[a\right]-s_{j}\left[a\right]\right|$$

In general, the global distance measures described above are susceptible to noise [11]. This is especially true in high-dimensional data because differences in irrelevant attributes can spuriously increase the distance between two samples that have the same values for the relevant attributes. Conversely, apparently nearby samples may in fact be quite distant when only the relevant attributes are considered. One way to mitigate the effect of irrelevant attributes is to weight the contribution of each dimension differently such that the global distance measure is influenced more by the higher weighted dimensions than by lower weighted ones. These dimension weights may be obtained from prior knowledge about the attributes or changed dynamically by using Relief’s own output. For example, Draper et al. developed Iterative Relief [12], in which Relief is applied repeatedly and during each iteration the dimension weights are set to the attribute weights obtained at the previous iteration.

#### Neighbor weighting function

**Figure 1:**
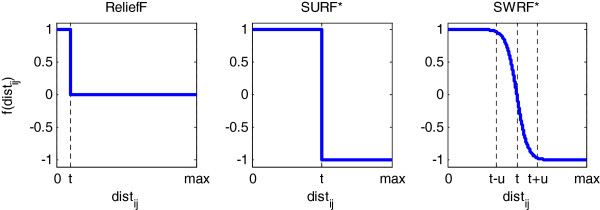
*Neighbor weighting functions for ReliefF, SURF*, and SWRF*. The x-axis for each plot denotes the distance of sample s_j_ from sample s_i_ and the y-axis denotes weight in the range [−1, 1]. Parameter t represents the function center and was set to the mean pairwise distance between samples. Parameter u is the function width and was set to the standard deviation of pairwise distances.

The neighbor weighting function denoted by *f*(*dist*_*ij*_) determines which neighbors count for how much in Relief’s update rule. Neighbor weights are determined as a function of *dist*_*ij*_, and take values from −1 to 1. A neighbor weight of 1 or −1 means that the neighbor influences the weight update maximally; a neighbor weight of 0 means that the neighbor does not influence the weight update at all. Several previously described variations of Relief differ essentially in the neighbor weighting function. For ReliefF, the neighbor weighting function is defined on a sample-wise basis, yielding a function *f*^*i*^ for each sample *i*:

$$\begin{array}{l} f_{\text{hit}}^{i}({\text{dist}}_{\mathrm{ij}})=\left\{ \begin{array}{c} 1\;\text{if}\;{\text{dist}}_{\mathrm{ij}}\leq t_{\text{hit}}^{i}\\ 0\;\text{if}\;{\text{dist}}_{\mathrm{ij}}>t_{\text{hit}}^{i} \end{array}\right.\\ f_{\text{miss}}^{i}({\text{dist}}_{\mathrm{ij}})=\left\{ \begin{array}{c} 1\;\text{if}\;{\text{dist}}_{\mathrm{ij}}\leq t_{\text{miss}}^{i}\\ 0\;\text{if}\;{\text{dist}}_{\mathrm{ij}}>t_{\text{miss}}^{i} \end{array}\right. \end{array}$$

where *t*^*i*^_*hit*_ is the distance of the *k*th nearest hit to *s*_*i*_, and *t*^*i*^_*miss*_ is the distance of the *k*th nearest miss to *s*_*i*_ (assuming no ties for the *k*th hit or the *k*th miss). For SURF*, the neighbor weighting function is a step function defined globally as a function of a single parameter:

$$f\left({\text{dist}}_{\mathrm{ij}}\right)=\{\begin{array}{c} 1\;\text{if}\;{\text{dist}}_{\mathrm{ij}}\leq t\\ -1\;\text{if}\;{\text{dist}}_{\mathrm{ij}}>t \end{array}$$

where *t* is a user defined distance threshold (e.g., mean pairwise sample distances). Figure 1 shows graphically the neighbor weighting functions used in ReliefF and SURF*.

### Sigmoid Weighted ReliefF Star (SWRF*)

**Figure 2:**
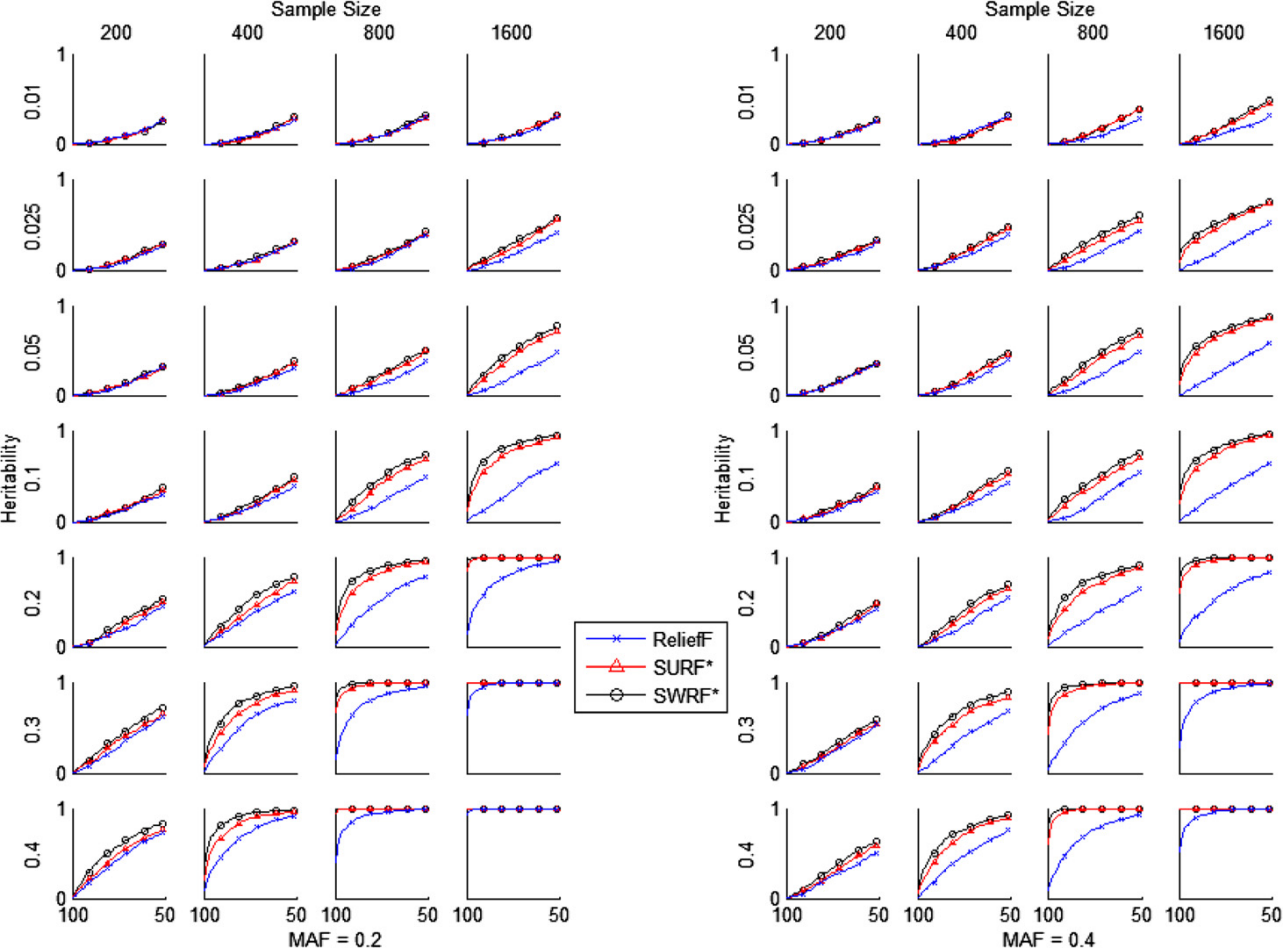
Plots comparing the success rates of ReliefF, SURF*, and SWRF*. Algorithms are compared across a range of sample sizes, heritabilities, and minor allele frequencies (MAFs). The x-axis for each plot denotes cutoff percentile and the y-axis denotes success rate.

We now describe a new variation of ReliefF called SWRF*. In the MoRF framework, SWRF* differs from ReliefF and SURF* in that it uses a sigmoid neighbor weighting function that is defined as:

$$f\left({\text{dist}}_{\mathrm{ij}}\right)=\frac{2}{1+e^{\frac{-\left(t-{\text{dist}}_{\mathrm{ij}}\right)}{u/4}}}-1$$

where *t* and *u* are parameters that define the center and width of the sigmoid function and *dist*_*ij*_ is the Hamming distance. The value 4 scales the width *u* and was chosen as described in the next paragraph. This neighbor weighing function takes values close to 1 and −1 for nearby and distant neighbors respectively, and provides a smooth transition of weights for the intermediate neighbors. The star in SWRF* indicates that it uses both nearby and distant neighbors, similar to SURF*. In our experiments, we set *t* to the mean pairwise distance between samples, and *u* to the standard deviation of the pairwise distances. Figure 2 depicts this neighbor weighting function.

The motivation for using the sigmoid function in SWRF* compared to the step function in SURF* is to avoid the large change in weight that may occur due to a small change in distance as can happen with samples that are close to the distance threshold *t*. The width of the sigmoid function is scaled by a factor of 4. The value of 4 provided the best performance among a range of values that included 1, 2, 3, 4, 6, 8, and 16. We evaluated the candidate values for the scale factor using 50 replications of the 200 and 400 sample size datasets. If the scale factor is large (>16) the function becomes narrow and behaves like a step function. Less than 20% of the samples are given weights significantly different from SURF*, and the performance of SWRF* is similar to SURF*. If the scale factor is small (<1), the function becomes too wide and assigns less than 1% of the samples the “full” weight (|*f(x)|* > 0.95). The sigmoid function loses it characteristic plateau regions, and behaves like a ramp function. A scale factor of 4 assigns full weight to sample pairs that are more than roughly one standard deviation from the pairwise distance mean. This yields an intermediate number of neighbor pairs (~30%) which are assigned full weight.

### Evaluation of SWRF*

The success rates of ReliefF, SURF*, and SWRF* on synthetic data are plotted in Figure 2. The algorithms were evaluated on their ability to identify the two epistatic SNPs out of a set of 1000 noisy variables across multiple genetic models and replications. Each plot gives the success rate on 500 datasets with a specific sample size and heritability. The *x*-axis of each plot denotes the cutoff percentile (100^th^ to 50^th^ percentile in steps of 1 percentile point) for the rank-ordered list of the 1000 SNPs. The *y*-axis denotes the fraction of the 500 datasets in which the two epistatic SNPs are present in the list of rank-ordered SNPs above the specified percentile cutoff in the top ranked SNPs. At small heritabilities and sample sizes when the genetic effect is small all three algorithms performed poorly, while at large heritabilities and sample sizes when the genetic effect is large all three performed well. At intermediate heritabilities and sample sizes, SWRF* and SURF* performed significantly better than ReliefF, and SWRF* outperformed SURF*.

Table 1 gives the results of Fisher’s exact test comparing the success rates of SWRF* and ReliefF at the 95^th^ percentile cutoff (i.e., at *x-*axis value of 95 in Figure 2) for two MAFs (0.2 and 0.4), seven heritabilities (ranging from 0.001 to 0.4) and four sample sizes (200, 400, 800 and 1600). At the 0.05 significance level, SWRF* performed statistically significantly better than ReliefF for large heritabilities (>0.2) and sample sizes (>800). Table 2 shows the results of Fisher’s exact test comparing the success rates of SWRF* and SURF* at the 95^th^ percentile cutoff. At the 0.05 significance level, SWRF* performed statistically significantly better than SURF* at intermediate sample sizes (400 and 800) and intermediate to higher heritabilities (0.2, 0.3 and 0.4 at MAF = 0.2 and 0.1, 0.2, 0.3 and 0.4 at MAF = 0.4). In addition, SWRF* never performed statistically significantly worse than SURF* at any of the MAF, heritability and sample size settings that we tested.

**Table 1:** Fisher's exact test p-values comparing success rates of SWRF* and ReliefF at the 95^**th **^**percentile cutoff**

	**Sample size**				
MAF	Heritability	200	400	800	1600
0.2	0.001	1	1	0.3737	0.6242
0.2	0.025	1	0.0692	0.0346	**<0.0001**
0.2	0.05	0.2492	0.7255	0.4766	**<0.0001**
0.2	0.1	1	1	**<0.0001**	**<0.0001**
0.2	0.2	1	0.0091	**<0.0001**	**<0.0001**
0.2	0.3	0.0081	**<0.0001**	**<0.0001**	**<0.0001**
0.2	0.4	**0.0002**	**<0.0001**	**<0.0001**	0.0164
0.4	0.001	1	1	1	0.0640
0.4	0.025	0.1237	0.8020	**<0.0001**	**<0.0001**
0.4	0.05	1	0.7527	**<0.0001**	**<0.0001**
0.4	0.1	1	0.3546	**<0.0001**	**<0.0001**
0.4	0.2	0.5466	0.1433	**<0.0001**	**<0.0001**
0.4	0.3	0.1178	**<0.0001**	**<0.0001**	**<0.0001**
0.4	0.4	0.1433	**<0.0001**	**<0.0001**	**<0.0001**

Shaded cells show SWRF* outperforms ReliefF at the 0.05 significance level (Bonferroni corrected).

**Table 2:** Fisher's exact test p-values comparing success rates of SWRF* and SURF* at the 95^**th **^**percentile cutoff**

	**Sample size**				
MAF	Heritability	200	400	800	1600
0.2	0.001	1	1	1	1
0.2	0.025	1	0.7730	1	0.5123
0.2	0.05	0.2173	1	1	0.0087
0.2	0.1	1	0.4206	0.0385	0.0122
0.2	0.2	1	0.1307	**<0.0001**	0.0611
0.2	0.3	0.6852	**<0.0001**	**<0.0001**	1
0.2	0.4	0.0871	**<0.0001**	0.2874	1
0.4	0.001	1	1	1	1
0.4	0.025	1	0.6045	0.2231	0.0831
0.4	0.05	1	1	0.0922	0.0151
0.4	0.1	0.6242	0.4991	0.0229	**0.0004**
0.4	0.2	0.6866	0.3833	0.0235	**<0.0001**
0.4	0.3	0.2579	0.1199	**0.0002**	1
0.4	0.4	0.2881	**0.0002**	**<0.0001**	1

Shaded cells show SWRF* outperforms SURF* at the 0.05 significance level (Bonferroni corrected).

## Conclusions

Efficient identification of interactions in SNP data is an important and challenging computational problem. The Relief algorithms provide a promising first stage method for identifying disease associated SNPs large-scale genomic datasets, even in the presence of nonlinear interactions and small main effects.

We have developed a framework called MoRF that abstracts the common features of the Relief algorithms. Several recently developed variations of the Relief algorithm may be viewed as distinct specifications of this framework’s neighbor weighting function. Novel enhanced variations of ReliefF can be developed by specifying new component functions in MoRF.

Using the MoRF framework we developed a new variation of ReliefF called SWRF* that uses a sigmoid neighbor weighting function. SWRF* performed significantly better at identifying interacting genetic variants in synthetic data than ReliefF and the recently described method SURF*.

## Methods

We evaluated ReliefF, SURF*, and SWRF* on a large set of synthetic SNP datasets. For ReliefF, we set *k* = 10. For SURF*, we set the distance threshold *t* to the mean pairwise distance between samples. For SWRF*, we set sigmoid function parameters *t* and *u* to the mean pairwise distance and to the standard deviation of the mean pairwise distance, respectively.

We used synthetic SNP data that were used by Moore et al. in evaluating SURF* [10]. The data is available for download from http://discovery.dartmouth.edu/epistatic_data/. The data were generated from 70 two-SNP epistatic models with different disease penetrance functions, minor allele frequencies (MAFs) of 0.2 or 0.4, and seven heritabilities ranging from 0.01 to 0.40 (0.01, 0.025, 0.05, 0.10, 0.20, 0.30, and 0.40) that might be expected for a common disease. To study the effect of sample size, from a given model, 100 datasets were generated for each of four sample sizes (200, 400, 800 and 1600) where each dataset contains equal number of disease and healthy samples. For a generated pair of epistatic SNP values, a set of 998 SNPs that were assigned random values was appended to simulate SNPs that are non-informative with respect to the disease status. In all, 28,000 datasets were analyzed.

We evaluated the performance of ReliefF, SURF*, and SWRF* on power. For a set of 500 datasets generated from a group of 5 related models, power is a number from 0 to 1 that denotes the proportion of datasets in which both epistatic SNPs are located in the list of rank-ordered SNPs above a specified cutoff. For example, in a dataset of 1000 SNPs, a cutoff of 95^th^ percentile implies that the top ranked 50 SNPs (top 5%) are examined for the presence of the two epistatic SNPs. To graphically compare the algorithms, we plotted the power of each algorithm for cutoffs varying from 100^th^ percentile to the 50^th^ percentile.

We also statistically compared the performance of SWRF* with that of SURF* and ReliefF at the 95^th^ percentile cutoff. To compare two algorithms at this cutoff, we compared the differences between their success rates using used Fisher’s exact test. For a set of 500 datasets generated from a group of 5 related models, the success rate is the number of datasets in which both epistatic SNPs are located in the list of rank-ordered SNPs above the 95^th^ percentile cutoff. This value is represented by the points on the Figure 2 curves corresponding to *x*-axis values of 95. We considered a result to be statistically significant when *p* < 0.05, and we applied the Bonferroni correction in order to account for multiple hypothesis testing (we conducted a total of 56 Fisher’s exact tests).

The algorithms presented in this paper were implemented in the C# programming language. The program source code and compiled executables are available for download at the open source code repository GitHub ( http://www.GitHub.com, username MattStokes42, project MoRF).

## Competing interests

The authors have no competing interests.

## Authors’ contributions

SHV conceived the study; MES developed, implemented and evaluated the algorithms. Both authors read and approved the final manuscript.
